# Safety and efficacy of a novel ‘One-Visit, Both-Cataracts’ high-volume see-and-treat immediate sequential bilateral cataract surgery service in a public healthcare setting

**DOI:** 10.1038/s41433-025-03659-8

**Published:** 2025-02-09

**Authors:** Maher Alsusa, Shakeel Ahmad, Zoe Smith, Emma Tutchings, Sam Evans, Elizabeth Wilkinson, Harry Roberts

**Affiliations:** 1 University of Exeter Medical School, Exeter, UK; 2 West of England Eye Unit, Royal Devon University Healthcare NHS Foundation Trust, Exeter, UK; 3 Lutra Health Ltd, Pencoed, UK; 4 Getting It Right First Time (GIRFT), NHS England, London, UK; 5 Faculty of Health and Life Sciences, University of Exeter, Exeter, UK

**Keywords:** Health services, Outcomes research

## Abstract

**Purpose:**

To evaluate the safety and efficacy of a novel cataract surgery pathway that combines a See-and-Treat (S&T) model with Immediate Sequential Bilateral Cataract Surgery (ISBCS) at the Nightingale Hospital, Exeter, UK.

**Methods:**

A retrospective observational study was conducted on 102 consecutive patients (204 eyes) who underwent S&T ISBCS between July 2023 and July 2024. Patients were triaged based on referral information and underwent preoperative telephone consultations. On the day of surgery, clinical assessment and bilateral cataract surgery were completed in a single visit. Data collected included patient demographics, intraoperative and postoperative outcomes, and complications.

**Results:**

Of the 127 patients listed, 102 (84.3%) completed S&T ISBCS. No intraoperative complications were recorded. Fourteen patients (13.7%) required unplanned postoperative consultations, with most cases being non-sight-threatening and self-resolving. Cystoid macular oedema (CMO) was reported in 2.9% of eyes, with no cases of visual loss or endophthalmitis.

**Conclusion:**

The S&T ISBCS model demonstrated safety and efficiency in delivering cataract care, with a high one-visit completion rate and low complication rates. This model offers significant time and resource savings whilst maintaining patient safety. It holds potential for broader implementation in healthcare settings facing increased demand for cataract services. Further studies are recommended to assess long-term outcomes and optimise this approach.

## Introduction

Cataract surgery is the most commonly performed surgical procedure in both the UK and worldwide, with demand projected to rise significantly due to an aging population [1, 2]. This increase poses substantial challenges for healthcare systems like the NHS, which is due to face a potential 50% rise in cataract-related costs in the coming decades [3, 4]. These pressures emphasise the urgent need for more efficient cataract care models.

Traditionally, bilateral cataract surgery in the UK has followed a Delayed Sequential Bilateral Cataract Surgery (DSBCS) model, requiring two separate hospital visits [5]. However, Immediate Sequential Bilateral Cataract Surgery (ISBCS)—where both eyes are operated on during the same session—has gained acceptance due to growing evidence supporting its safety and efficacy. ISBCS offers several benefits, including fewer hospital visits, faster recovery, and reduced costs. However, its adoption remains limited across the NHS [6–8].

To address clinical pressures and long waiting lists, the Nightingale Hospital in Exeter, UK, has implemented a ‘See-and-Treat’ (S&T) model over the past two years for low-risk cataract referrals. Under this model, patients receive written preoperative information and a telephone consultation with a consultant ophthalmologist or senior hospital optometrist. They then attend a single appointment for clinical assessment, investigations, and surgery, all within a staggered two-and-a-half-hour visit as part of an all-day high-volume cataract list. After uncomplicated surgery, patients are discharged and advised to follow-up with their optometrist for postoperative refraction. More recently, our service has combined the S&T model with ISBCS, offering patients the opportunity to receive bilateral cataract surgery with only a single visit to the hospital for their entire management.

To our knowledge, no published studies have evaluated the safety and clinical outcomes of a service that performs both assessments and bilateral surgery ‘One-Visit, Both-Cataracts’ in a single hospital visit.

This study aims to evaluate the safety, efficacy, and potential of this combined S&T and ISBCS model as a solution for high-volume, low-risk cataract management.

## Methods

### Study design

This retrospective observational study assessed outcomes following one-stop assessment and management of cataracts through S&T ISBCS, conducted at the Nightingale Hospital, Royal Devon University Healthcare NHS Foundation Trust (RDUH). The methods of this study adhered to the declaration of Helsinki. Approval for this audit was given by the RDUH Clinical Audit and Effectiveness Department (REF: 24-1428).

### See-and-treat pathway

**Fig. 1:**
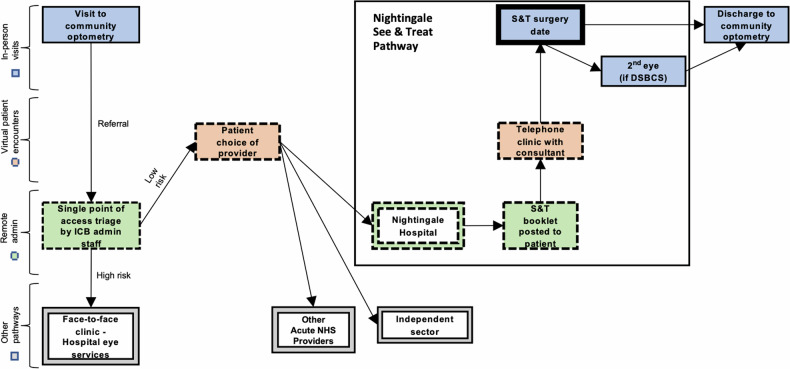
See-and-treat cataract surgery pathway, Nightingale Hospital, Exeter.

The S&T cataract surgery pathway is offered to new cataract referral patients deemed low-risk subsequent to screening the optometrists’ referral letter by a single point of access (SPoA) team (Fig. 1 – See-and-treat cataract surgery pathway, Nightingale Hospital, Exeter), (S&T at SpOA criteria – Supplementary Material 1). Patients deemed unsuitable for triage into S&T are offered a face-to-face cataract clinic appointment.

Patients eligible for S&T receive an information booklet by post or email and are signposted to the cataract decision tool by NHS England (Decision support tool: making a decision about cataracts – Supplementary Material 2). A telephone consultation will then be scheduled with a consultant ophthalmologist or specialist optometrist to conduct the preoperative telephone consultation. The structure of the content covered by this preoperative counselling can be seen in Table 1.

**Table 1:** Structure of the content covered within the preoperative telephone consultation, conducted by a consultant ophthalmologist or specialist optometrist.

Structure of Preoperative Telephone Consultation
• Confirms patient details and agreement with content in referral letter
• Ensures patient has received, read and understood S&T cataract resources
• Establishes consent with S&T model
• Establishes symptoms and willingness for surgery
• Past ophthalmic history
• Discusses risks and benefits of cataract surgery
• Discusses refractive outcome options and documents a provisional refractive target
• If suitable for offering ISBCS, discusses options of ISBCS or DSBCS, the advantages/disadvantages of each and records patients’ preference.
• Discusses that if additional conditions are discovered on the day of surgery, they may not be eligible for surgery on one/both eyes.
• Any additional questions arising from the S&T booklet or from the phone call are answered.

Following the phone call, the patient received written correspondence relating to the telephone consultation, and subsequently were offered an individual timeslot to attend the Nightingale Hospital for S&T surgery. Patients preferring ISBCS were informed that final suitability would be determined on the day of surgery. If unsuitable for ISBCS, an alternate plan would be made which may include DSBCS or unilateral surgery. Exclusion criteria for ISBCS were derived from Royal College of Ophthalmologists’ (RCOphth) guidelines, detailed in Table 2 [1].

**Table 2:** Exclusion criteria for offering ISBCS, derived from RCOphth guidelines.

Exclusion criteria for offering ISBCS
• Insufficient contralateral cataract/unilateral pseudophakia
• Lives alone/lacks sufficient care in home environment
• Presence of ophthalmic conditions increasing risk of postoperative complications or refractive surprise, such as (but not limited to):
○ Corneal oedema (significant Fuchs endothelial dystrophy, previous keratoplasty)
○ Postoperative uveitis (significant uveitis history)
○ Cystoid macular oedema (significant epiretinal membrane/vitreomacular traction/retinal vascular disorders or significant diabetic retinopathy)
○ Refractive surprise (corneal dystrophies or significant keratoconus)
○ Increased risk of endophthalmitis (significant blepharitis, poor personal hygiene/neglect)

### Day of surgery assessment

**Fig. 2:**
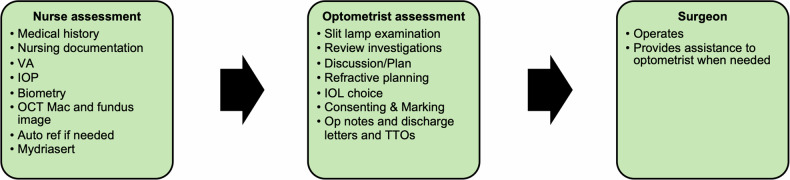
Day of surgery S&T processes (VA = Visual Acuity, IOP = Intraocular Pressure, OCT = Optical Coherence Tomography,  IOL = Intraocular Lens, TTO = [Medicines] To Take Out).

On the day of S&T surgery, patients underwent assessments by nurses and specialist optometrists (Fig. 2 – Day of surgery S&T processes). Visual acuity was measured with logMAR, intraocular pressure with iCare (iCare Oy, Vantaa, Finland) and biometry with the IOL Master 700 (Carl Zeiss Meditec AG, Jena, Germany). Optical coherence tomography and fundus imaging were performed with Cirrus Photo OCT/fundus camera 600 (Carl Zeiss Meditec AG, Jena, Germany).

### ISBCS

As per ISBCS requirements, each eye was treated as a separate procedure using distinct sets of surgical instruments and materials, with unique batch numbers for each. After successful surgery on the first eye, patient consent was verbally reconfirmed by the surgeon for the second eye, and the surgeon rescrubbed for the procedure.

Patients were treated on high-volume theatre lists with 10 cases routinely booked to each half day list. ISBCS was performed by 2 experienced surgeons (HR/SA) with low rates of complications evidenced by regular audit. Cataract surgery was performed using the Stellaris phacoemulsification machine (Bausch & Lomb, Rochester NY, USA) and the default intraocular lens was the Tecnis DCB00 (Johnson and Johnson, New Brunswick NJ, USA). Sterile air flow was provided by a SurgiCube (SurgiCube International BV, Zuidland, Netherlands). Default postoperative therapy was Maxitrol® (Novartis Pharmaceuticals, Basel, Switzerland) four times a day for 28 days.

### Data collection

Participants included in this retrospective analysis were the first 102 consecutive patients who underwent S&T ISBCS at the Nightingale Hospital. Data was extracted through retrospectively accessing electronic patient records (Epic, Epic Systems Corp., Verona WI, USA). Individual data retrieved from records include date of birth, sex, and date of surgery. Management-related data extracted includes: reasons for cancellation/conversion to DSBCS or unilateral, any intraoperative complications, details of any scheduled or unplanned postoperative visits as well as any postoperative complications. Of those experiencing a postoperative complication, details of further interventions as well as final visual acuity compared with baseline were recorded.

## Results

**Fig. 3:**
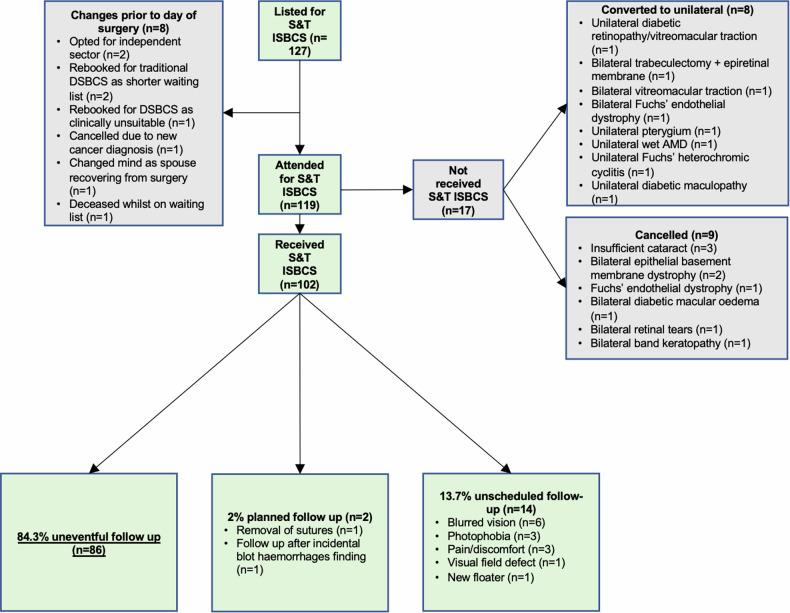
Patient attendances and outcomes following initial listing for S&T ISBCS, including one-visit completion rate and reasons for reattendance.

127 patients were originally listed for S&T ISBCS. Eight patients (6.3%) exited the pathway between listing and the day of surgery, and 17 (13.4%) were deemed unsuitable for ISBCS on the day of assessment. Of these, nine (7.1%) required alternative management, whilst eight (6.3%) underwent unilateral surgery or DSBCS. A flow diagram of patient outcomes is shown in Fig. 3.

A total of 102 consecutive S&T ISBCS procedures (204 eyes) were performed at the Nightingale Hospital, between 27/07/2023 and 16/07/2024. The cohort included 54 males (52.9%) and 48 females (47.1%). The mean age was 74.4 (±7.45) years. All operations were uncomplicated and in no cases was the surgery abandoned after the first eye. All operations were performed on specific high-volume theatre lists with 10 eyes booked per half day session. Data collection was performed up to 16/10/2024 to ensure a minimum of three months had lapsed for adequate inclusion of any postoperative complications.

### One-visit completion rate and scheduled returns

A total of 86 out of 102 patients (84.3%) completed the one-visit-only pathway and were discharged to the community without any unplanned or scheduled returns to the hospital. One patient (0.9%) required a scheduled postoperative visit for corneal suture removal. Another patient (0.9%) was referred to the retinal diagnostics clinic for a scheduled postoperative visit due to incidental findings of blot haemorrhages on the day of surgery.

### Postoperative outcomes

Unexpected postoperative visits were necessary for 14 patients (13.7%) following the patient contacting the department. Presenting complaints included blurred vision (*n* = 6), pain/discomfort (*n* = 3), photophobia (*n* = 3), new floaters (*n* = 1), visual field defects (*n* = 1).

Subsequent diagnoses were overwhelmingly self-resolving and included a range of non-sight threatening causes. Acute uncomplicated posterior vitreous detachment was observed in one eye (0.49%). Besides ‘safety netting,’ no additional intervention was needed. Maxitrol toxicity was observed in seven eyes of four patients (3.4%) and treatment was amended to preservative-free dexamethasone 0.1% in such instances. Self-resolving corneal oedema occurred in four eyes of two patients (2%), of which both patients self-presented within the first three days postoperatively and were found to have an appropriate examination given the recency of surgery. One patient had a reassuring examination and was diagnosed with unilateral negative dysphotopsia (0.49%). Overall, more than three-quarters of patients (11 patients, 78.6%) self-presenting back to the service were found to have a generally reassuring examination without the need for significant intervention.

### Postoperative complications

Regarding significant postoperative complications, cystoid macular oedema (CMO) was observed in six eyes of four patients (2.9%), three of whom self-presented back to the service, with the remaining case referred in following a sight test with the community optometrist. All were initially treated with topical dexamethasone and ketorolac. Three out of six eyes (1.5%) required no further treatment. One eye (0.49%) had a further course of Nepafenac and prednisolone acetate drops after which the CMO resolved.

Two eyes (0.98%) required sub-Tenon’s triamcinolone acetonide (Kenalog®, Bristol Myers Squibb, Princeton NJ, USA) followed by intravitreal dexamethasone implants (Ozurdex®, Allergan Inc., Irvine CA, USA).

No cases of retinal detachment, endophthalmitis, toxic anterior segment syndrome, refractive surprise or the need for further surgery were recorded. No cases of moderate (≥0.3 logMAR) or severe (≥0.6 logMAR) visual loss were observed.

## Discussion

Since the advent of phacoemulsification, we have observed ever-improving safety and clinical results. In the UK public sector amongst others, this has led to a trend in the re-organisation of the processes before and after cataract surgery. Over the years, the day one postoperative review has been abandoned, followed by any hospital based follow-up entirely for the majority of patients [9–11]. Some services have also moved towards pathways with various names whereby good-quality referrals of low-risk patients are triaged into an accelerated pathway [12–14]. NHS Trusts are universally encouraged to offer specific ‘high-volume, low-complexity’ theatre lists with a minimum of 10 eyes per theatre list [15]. Finally, while the rates of ISBCS remain low in the UK, there is a general trend of increasing interest on an established evidence base of equivalent safety to DSBCS [2, 4, 7, 16]. We believe we have for the first time, developed a service which combines all of the above elements to uniquely offer our patients the option of having clinical assessment, bilateral cataract surgery and safe discharge all on the same day within only one trip to the hospital as part of a high-volume theatre list.

Patient advantages of ISBCS include fewer hospital visits, quicker visual rehabilitation, avoidance of anisometropia and shorter waiting times [2]. From a healthcare provider standpoint, ISBCS has demonstrated enhanced theatre productivity and more optimised resource management [17, 18]. Other benefits of ISBCS include a reduced carbon footprint and reduced patient anxiety resulting from fewer hospital attendances [2, 4]. National ISBCS tariffs stand at 185% of those from unilateral cataracts, incentivising its implementation [19]. ISBCS is supported both by the National Institute for Health and Care Excellence (NICE) and the RCOphth [20, 21]. Although the RCOphth has encouraged ISBCS to help tackle backlogs resulting post-pandemic [21], uptake is still limited as suggested by recent National Ophthalmology Database (NOD) data and represents less than 1% of all UK cataract surgery [3, 22].

The disparity between the patient acceptance of ISBCS and its presence within cataract services nationwide is profound. Over half of participants in a London study [2], and 71.6% in our own population [23] preferred ISBCS, whilst recent data suggests approximately 0.5% of bilateral cataract operations nationally are immediately sequential [3]. However, the presence of ISBCS is increasing across trusts nationwide. Recently, ISBCS was implemented at Buckingham Healthcare NHS Trust (BHT), where 10.7% of all cataract operations across a one-year period were ISBCS [4]. Our cohort of 204 S&T ISBCS eyes represent 4.7% of all cataracts treated by the RDUH trust over the same time period (*n* = 4308). These results tangibly demonstrate the feasibility and success of offering ISBCS to a growing cohort of patients. The established efficiency benefits of ISBCS are further compounded when the pre-operative assessment is combined in the same visit, while simultaneously providing a convenient patient-centred approach with high levels of satisfaction.

Of the 17 patients who attended but did not receive ISBCS, 47.1% (*n* = 8) were still treated for one eye after identifying unprecedented ocular pathology precluding safe ISBCS on the day of surgery. Cancellations only accounted for 7.6% (*n* = 9) of all S&T attendances, of which all were due to clinical conditions detected at the time of the assessment. A certain rate of changing the management plan on the day is inevitable given the nature of the S&T service which is being run and applies to both the DSBCS and ISBCS patients. Patients on our S&T pathway are advised in writing before the day of surgery to expect a possibility of a change in management plan on the day. The rates of change in management plan are relatively low but could in future be factored in to how many patients are booked onto the list, or alternatively have local patients on standby to take the place of any cancellations.

Guidance from RCOphth advises against consenting on the day of surgery for one-stop ISBCS [1], however our consent process begins with the issuing of information to the patient and signposting to online resources prior to a telephone consultation with a consultant or optometrist. This ensures that patients are fully informed and consented prior to the day of surgery and merely confirm consent with a physical signature on the day. In the future we are moving towards the patient consenting electronically from home following the telephone consultation (MyChart, Epic Systems Corp., Verona WI, USA).

Traditionally, early postoperative review following cataract surgery was the standard, typically involving ‘day one’ reviews requiring an extra hospital attendance. However, evidence has suggested no further benefit is elicited through early review post-cataract surgery in low-risk patients with uneventful surgery [24]. Since the nature of S&T ISBCS means only low-risk patients are triaged into the pathway, discharging to community imminently post-cataract surgery remains a safe option [11]. With our service, the safety and efficacy of discharge to the community is built upon careful selection of patients to be offered S&T ISBCS, accuracy of optometry referrals, and ‘safety netting’ advice to patients to contact the department should concerns arise. In turn, unnecessary postoperative hospital attendances will be minimised without increased risk to patient safety or visual outcomes, saving valuable time and resources to be used for new referrals.

Both our intraoperative and postoperative rates of significant complications are below nationally reported rates and in the literature. National rates of post-cataract surgery vision loss stand at 0.48% [25], in comparison to our 0% rate of vision loss. A large retrospective analysis of 81,984 eyes found incidence of post-cataract surgery CMO to be 1.17% [26]. Moreover, a reported rate of CMO within a cohort of 9776 patients retrospectively analysed stood at 1.44% [27], similar to the recently published NOD rate of 1.4% [25]. A key limitation of these larger database studies is likely to be the underreporting and documentation of such complications. Our rate of 2.9% reflects a low incidence of CMO post-S&T ISBCS, in keeping with or lower than similar studies, for instance a UK based ISBCS study and the European Society of Cataract and Refractive Surgeons PREMED study, which had CMO rates of 4.3% and 3.4% respectively [28, 29].

### Limitations

All ISBCS operations were performed by only two surgeons, potentially hindering the applicability of our results to other surgeons. Furthermore, postoperative refraction data is not available due to lack of a service level agreement between our trust and local community optometrists to receive postoperative data, meaning refractive outcomes cannot currently be assessed. Cataract services throughout our whole NHS trust, not just at the Nightingale Hospital, are currently unable to return information to the NOD but steps are being taken to remedy this. In keeping with guidelines from ‘Getting It Right First Time’ (GIRFT) [19], routine uncomplicated cataract operations were safely discharged to the community with no hospital follow-up, however this may mean that our observed complication rate was under-reported. Whilst our complication rates were below or similar to published figures [3, 25–27], this study was designed as a proof-of-concept and therefore not sufficiently powered to detect differences between complications rates of rarer complications such as CMO, rhegmatogenous retinal detachment, endophthalmitis or suprachoroidal haemorrhage. Such outcomes are to be investigated elsewhere in the literature as they fall beyond the scope of this study.

## Conclusions

Advisory bodies including RCOphth, NICE and GIRFT advocate for both high-flow cataract pathways and also ISBCS. Our results have demonstrated the effectiveness of offering the assessment and management of both cataracts in a single hospital visit. S&T ISBCS ‘one-visit, both-cataracts’ offers multilevel benefits to all stakeholders, providing a safe, high quality and patient-centred service while reducing hospital visits, waiting times and costs of surgery. Based on our findings we encourage further adoption of this model in the health service.

## Summary

### What was known before

- Cataract care demand is anticipated to rise in the coming decades.
- There is a growing body of evidence supporting the implementation of ISBCS.
- Low-risk accelerated pathways within cataract care have operated successfully.

### What this study adds

- Combining See-and-Treat with ISBCS is a safe and effective approach to treating bilateral cataracts based on our findings.

## Supplementary information

Supplementary Material 1 - S&T at SPoA low risk criteria

Supplementary Material 2 - Decision support tool: making a decision about cataracts

## Data Availability

Data available upon written request to the corresponding author.
