# Supplementary material for: Safety and efficacy of a novel ‘One-Visit, Both-Cataracts’ high-volume see-and-treat immediate sequential bilateral cataract surgery service in a public healthcare setting
Source: Eye (Lond). 2025 Feb 9;39(7):1414–9. doi: 10.1038/s41433-025-03659-8 (PMC12044009; doi:10.1038/s41433-025-03659-8)
Supplement: Supplementary file 1 — Supplementary Material 1 - S&T at SPoA low risk criteria [file 41433_2025_3659_MOESM1_ESM.pdf]

CATEGORY *(just for info)*	**QUESTIONS**	***SUPPORTING INFO FOR PCFs***
AGE	1. 18+	*Patients must be 18 and over*
COMMUNICATION	2. Do you require any communication support? For example, do you need an interpreter, do you have severe dementia, or do you have severe learning difficulties?	*Additional comms support not typically provided*
MOVEMENT + MOBILITY	3. Would you struggle to lie flat and still for half an hour? For example, do you have a head tremor or severe claustrophobia?<br>4. Do you need more than 1 stick to get about?	*Patient must be able to lie still during the procedure.*<br><br>*Lots of walking to get to pre-op room and theatres and not much space, so patient must be ambulatory*
	5. Standard DRSS BMI question (BMI >40)	*No hoists available and possible weight limitations on couches, so BMI must be ≤40*
FITNESS + COMPLEXITY	6. Do you need a general anaesthetic<br>7. Do you need continuous oxygen<br>8. Have you had a cardiac arrest, heart attack or stroke in the last 6 months, or do you have an implantable defibrillator?<br>9. Are you under the care of the hospital for any other eye condition? Has your eye consultant ever suggested	*GA not available at Hubs/ISPs, patient will know if they absolutely must have a GA.*<br>*An implanted defibrillator (which is not ok) is different to a pacemaker (which is ok)*<br>*Patients with other ocular conditions (glaucoma, WET/DRY AMD etc)* ***may*** *be more complex and their usual consultant may have advised their cataract surgery takes place at the acute site. This will*

	your cataract surgery would be difficult or should be done in the eye hospital<br>10. Do you wear contact lenses?	*supersede patient choice on safety grounds.* ***If under a consultant already for another eye condition, we recommend you stay with this hospital so they can monitor both conditions, for continuity of care.***
